# Voltage-Induced Wrinkle Performance in a Hydrogel by Dielectric Elastomer Actuation

**DOI:** 10.3390/polym10070697

**Published:** 2018-06-22

**Authors:** Chao Tang, Bo Li, Chenbang Zou, Lei Liu, Hualing Chen

**Affiliations:** 1 School of Mechanical Engineering, Xi’an Jiaotong University, Xi’an 710049, China; wstcsues@163.com (C.T.); zouchenbang@stu.xjtu.edu.cn (C.Z.); aist456@163.com (L.L.); hlchen@xjtu.edu.cn (H.C.); 2 Shaanxi Key Laboratory for Intelligent Robot, Xi’an Jiaotong University, Xi’an 710049, China; 3 State Key Laboratory of Fluid Power and Mechanic Systems, Zhejiang University, Hangzhou 310027, China; 4 State Key Laboratory for Strength and Vibration of Mechanical Structures, Xi’an Jiaotong University, Xi’an 710049, China

**Keywords:** hydrogel, wrinkle, actuation, dielectric elastomer, optics

## Abstract

Hydrogel is a type of soft smart material and is responsive to stimuli. In the development of actuation in hydrogel, electrical actuation features a fast and universal strategy which is favored in the engineering system. Due to the difficulty in direct electrical actuation in hydrogel, in this study, an indirect actuation was attained via a dielectric elastomer. An aligned wrinkle pattern was obtained in the hydrogel upon a direct-current voltage, and it is reversible. The morphology and nonlinear mechanics of the electro-wrinkling deformation was characterized and analyzed. The optical property of the wrinkle in hydrogel was investigated, demonstrating a tunable blurring effect in optics. The electro-wrinkling performance offers a potential application with soft and tunable optical property in hydrogel-based actuators.

## 1. Introduction

Hydrogel is a type of soft materials, consisting of long-chain polymer networks and diverse liquid solvents. Owing to its large liquid component, a piece of hydrogel can deform largely, in terms of volume change by 50 times after swelling [1] or the attainable maximum stretch of 22 times in pure shear configuration [2]. This unique mechanical property offers promising applications in tissue engineering [3], soft robots [4] and biomimetic systems [5].

Hydrogel can be responsive if subjected to different stimuli, including pH value [6], temperature [7], humidity [8], light intensity [9], and pneumatic pressure [10]. Then, the hydrogels may form crease patterns, if constrained by a mechanical boundary layer [11], which can be developed as an active surface [12]; or hydrogel can generate a bending locomotion as a worm-like robot [13], as well as a non-invasive gripper [14]. However, these actuation strategies are sensitive to the environment and are only applicable in selected application cases. Pneumatic actuation is direct and easy to control, but requires complex air chamber design with an extra air pumping source. Thus, a more universal strategy for hydrogel in actuation is expected.

Electrical voltage or current is well-recognized and is the most adapted physical quantity in the mechatronic system, requiring a cable only for fast, clean and long distance transmission. Yet, an electrically activated hydrogel is more favorable. Owing to the ions in hydrogel, directly applying voltage on hydrogel may cause a local chemical instability or even breakdown [15]. So in this study, we present a new actuation route for the hydrogel via the electromechanical deformation of a dielectric elastomer.

Dielectric elastomer is able to generate large mechanical stretch under voltage. The voltage-triggered deformation in dielectric elastomer has been explored in various actuators for direct stroke [16] or motion output [17]. Recently, dielectric elastomer is a candidate for reversible and electrical-stretching platform on the micro-scale. Under this spotlight, a millimeter-wave phase shifter has been assembled with two dielectric elastomers in coordination [18]. Another example is a dielectric elastomer based soft manipulation for in vivo cell stretching [19]. These reports inspired us to use the dielectric elastomer to stretch a hydrogel electrically. Herein, the actuation stretch of the hydrogel with dielectric elastomer as well as the wrinkling morphology is demonstrated. The optical performances in transmission are characterized, illustrating a new application in soft and stretchable optics.

This manuscript is organized as follows. Section 2 introduces the deformation mechanism of the dielectric elastomer. Section 3 describes the experiments and the result. An electromechanical theoretical analysis is developed in Section 4, with calculation result. Section 5 extends the model to study the optical properties, accompanied by an illustration in electrical blurring effect. Section 6 concludes the results with discussion and prospects.

## 2. Deformation Mechanism of Dielectric Elastomer

**Figure 1:**
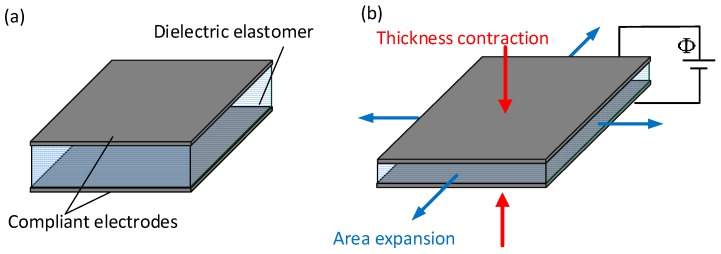
Schematic of an actuated dielectric elastomer (**a**) Reference state; (**b**) Actuation state.

A dielectric elastomer is an insulating material in a membrane shape. When the voltage in the order of kV is applied, positive and negative charges are accumulated separately on each surface of the dielectric elastomer. Due to the attraction of charges, the electrostatic force will compress the dielectric elastomer, and this soft and elastic material deforms in terms of thinning in thickness, as shown in Figure 1. The main factor which caused the deformation of the dielectric elastomer is Maxwell stress. The principal Maxwell stress is defined as

(1) $$P_{Maxwell}=\varepsilon_{0}\varepsilon_{r}{\left(\frac{V}{h}\right)}^{2}$$

where, $\varepsilon_{0}$ is the permittivity of free space, $\varepsilon_{r}$ is the relative permittivity, V is the applied voltage, and h is the real membrane thickness.

From the Equation (1), we can see that an increase in the actuation voltage or a decrease of the initial thickness of the dielectric elastomer is an effective method to improve the actuation of the dielectric elastomer actuator. Therefore, the dielectric elastomer membrane usually needs a mechanical pre-stretch to obtain a stabilized large actuation [20,21].

## 3. Experimental and Results

### 3.1. Hydrogel Synthesis

**Figure 2:**
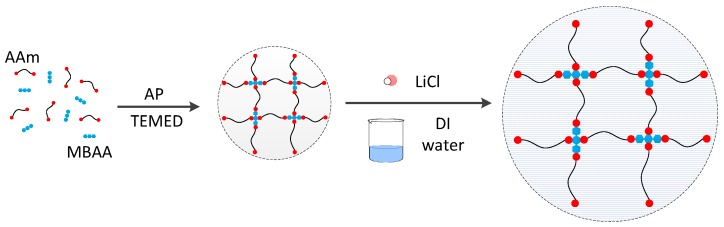
The synthesis of the hydrogel.

The hydrogel was synthesized, as shown in Figure 2. With reference to the research of conductive gel [22], acrylamide (AAm, A8887, Sigma-Aldrich, Saint Louis, MO, USA) was used as the monomer, *N*,*N*-methylenebisacrylamide (MBAA, M7279, Sigma-Aldrich, Saint Louis, MO, USA) was the crosslinkers, *N*,*N*,*N*′,*N*′-tetramethylethylenediamine (TEMED, T7024, Sigma-Aldrich, Saint Louis, MO, USA) was the crosslinking accelerator, and LiCl (V900067, Sigma-Aldrich, Saint Louis, MO, USA) was the electrolyte. AAm and LiCl were dissolved in deionized water at the concentrations of 2.2 and 3.3 M, respectively. MBAA was then added as 0.0006 wt % the weight of AAm. After degassing in a vacuum chamber, TEMED 0.0025 to the weight of AAm was added. The solutions were poured into a 150.0 × 150.0 × 0.1 mm^3^ glass mold. The gels were cured at room temperature for 90 min and cut into the designed shape using a laser cutter.

### 3.2. Hydrogel and Dielectric Elastomer in Actuation

**Figure 3:**
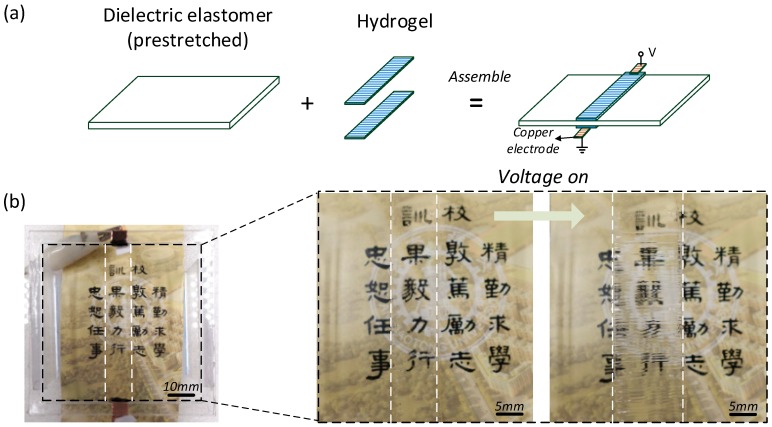
Hydrogels are attached to pre-stretched dielectric elastomer, consisting of a soft and fully transparent tri-layer laminate. After applying voltage, the hydrogel deforms and wrinkles. (**a**) Fabrication process of the actuator. (**b**) Blurring effect in optics. The white dash box showing the area which is covered with hydrogel.

The VHB membrane was stretched to four times their initial length and width and was fixed to a square-shaped acrylic frame. The inner side length of the square acrylic frame is 60 mm. Two pieces of hydrogel were attached to the pre-stretched dielectric elastomer (VHB 4910, 3M company, Sao Paulo, MN, USA), consisting of fully transparent tri-layer laminates, shown in Figure 3. The length and width of the hydrogel is 60 mm × 10 mm. Two copper electrodes which placed on the frame linked the hydrogel electrodes and the voltage amplifier. Hydrogel also serves as a compliant electrode, owing to LiCl electrolyte solution. Thus, the area in the dielectric elastomer covered by hydrogel can deform with the voltage on. So it is defined as electro-active; while the rest of the dielectric elastomer cannot be electrically actuated, so it is defined as the passive part providing mechanical boundary force. A signal generator (No. DG4062, RIGOL Agilent^TM^, Santa Clara, CA, USA) and a voltage amplifier (No. 1010B-HS, Trek^TM^, Amplifier, New York, NY, USA) generate an incremental voltage with a step of 20 V to the dielectric elastomer until the electrical breakdown occurs. The experimental setup is illustrated in the Supplementary materials (SM).

**Figure 4:**
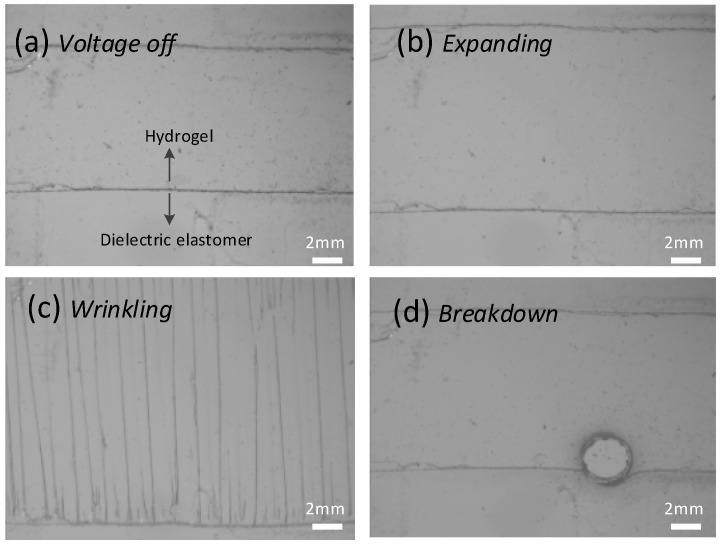
Actuation and wrinkling of the hydrogel via a dielectric elastomer. (**a**) Without a voltage, it is flat. (**b**) With a low voltage, the gel and elastomer expand. (**c**) Upon a critical voltage, the gel wrinkles. (**d**) As the voltage ramps, the actuator fails at breakdown.

The dielectric elastomer that is covered with hydrogel gel is defined as the electro active part. When subjected to voltage, the electro active area expands. Upon the expanding, it meets the rigid boundaries of the acrylic frame, the hydrogel with the dielectric elastomer wrinkles, tuning its transparency and showing a blurring effect in optics, as shown in Figure 3. Figure 4 collects the snap-shots of the process in the actuation using a microscope (No. SZX16, OLYPUM^TM^, Tokyo, Japan). After the voltage is turned on, the dielectric elastomer expands and drives the hydrogel to expand as well. Then upon a critical voltage, the hydrogel wrinkles by forming a regular patterned morphology. As the voltage ramps up further, the dielectric elastomer thins down consequently; so that it withstands an even higher electrical field which will finally causes the electrical breakdown failure. So as long as the voltage is below the breakdown criteria, the hydrogel is able to quickly change its transparency by forming wrinkles and can be used in optical applications.

The actuation deformation of the hydrogel experiences a transition from in-plane expansion to out-of-plane deflection via dielectric elastomer under a voltage [23]. Without a designed shape of actuation area, the out-of-plane deflection would be quite irregular and randomly patterned, since the imperfection in a local spot will disorder the wrinkle pattern. Buckled, crumpled, and wrinkled pattern have been identified using a circular-shaped electrode [24,25]. Aligning and tuning the wrinkle morphology has great potential in optical application, especially the property of transparency. Therefore, a rectangular-shaped hydrogel is presented here.

### 3.3. Wrinkle Morphology Characterization

**Figure 5:**
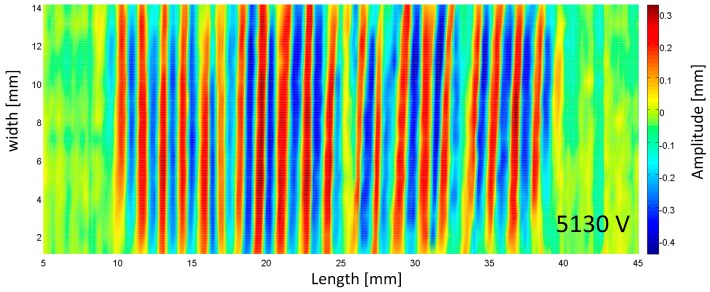
The characterization of the wrinkled hydrogel surface.

**Figure 6:**
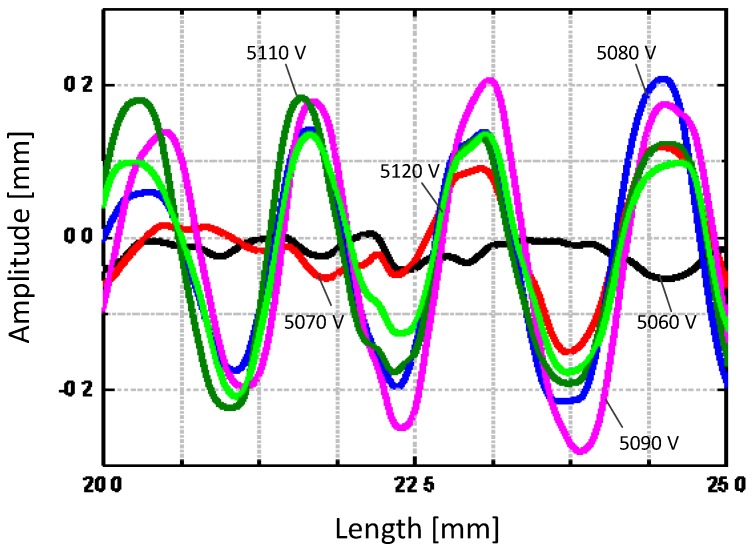
The relationship of wrinkle morphology as a function of the voltage.

**Figure 7:**
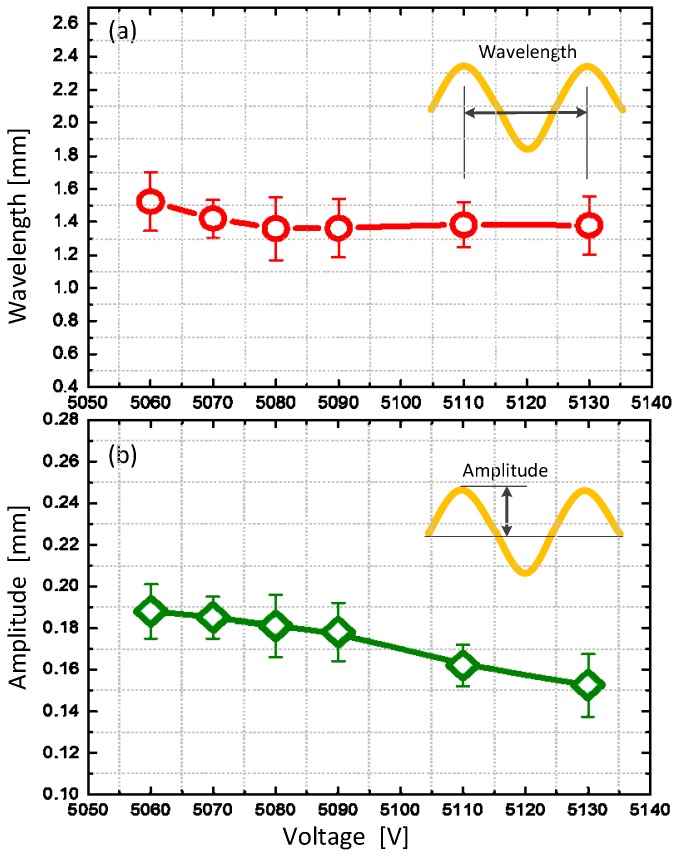
Voltage tuned wrinkling morphology of the hydrogel in (**a**) wavelength and (**b**) amplitude.

The morphology of the wrinkle surface was measured to show its out-of-plane displacement, as can be observed in the experimental setup in Figure S1 of SM. Figure 5 shows the morphology of the wrinkled hydrogel under a voltage of 5130 V. The wrinkles are in parallel along the width direction. The wavelength and depth of the wrinkle are consistent throughout the surface, indicating there is no global buckling. Figure 6 shows the growth of the wrinkle amplitude as a function of the voltage. The hydrogel is flat until a critical voltage, then it wrinkles, and evolves its pattern as the voltage ramps up. By examining the experiments, the change in wavelength and amplitude were characterized in statistics. By accounting for all the wrinkles in the whole membrane, the average value of the amplitude and the wavelength are calculated with error bar showing the variance. The overall tendency in the waveform vs. the voltage is shown in Figure 7. As the voltage increases, hydrogel maintains its level of wavelength, but its amplitude decreases slightly. Compared with the wrinkle in elastomer, different wrinkle patterns can be switched if the strain ramps up [26], but in our experiment, we observed only one type of wrinkle pattern with a slight change in its amplitude. If the voltage ramps up, the electrical breakdown has occurred, leading to the fundamental failure.

This is due to the nonlinearity in the mechanical property of hydrogel. In our experiment, the propagation of the wrinkle was recorded, as shown in Figure S2 in the SM. The hydrogel and dielectric elastomer deform with phase coexistence: The wrinkled part and flat part coexist at the same level of voltage. With the increasing voltage, the area of the wrinkled part expands at the expense of the flat part; therefore, the wrinkle propagates.

### 3.4. Optical Transmission Measurement

**Figure 8:**
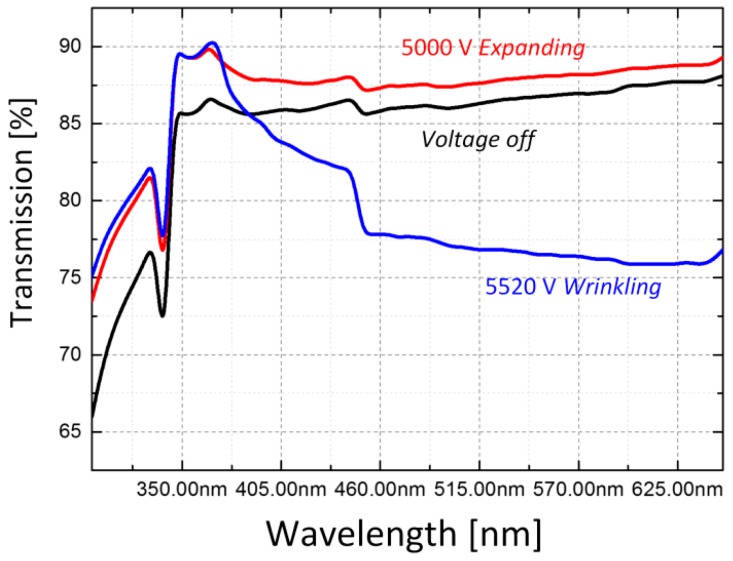
The optical transparency in the hydrogel is actuated by the voltage from flat to wrinkled state and transmission visible spectra are measured in the three actuation states.

The optical property is changed in the demonstration of the blurring effect on background Chinese characters, which can be found in Figure 3. The light transmission was measured in the visible light spectrum using a spectrophotometer, as shown in Figure 8. It is interesting that the hydrogel is even clearer, (high transmission and low absorbance) in the actuation before the wrinkled state. According to the Beer-Lambert law [27], when the hydrogel expands with the elastomer, the thickness decreases, promoting the transmission. However, in the wrinkled state, light travels in the gel and elastomer through complex reflection and refraction, suppressing the transmission.

Using the hydrogel electrode, we proposed a new route for visible light manipulation to achieve a soft and wearable camouflage by voltage tuning. The VIDEO (Video S1) is shown in the SI. In the video, the applied voltage is of 5200 V amplitude and 1 Hz frequency.

## 4. Theoretical Analysis

### 4.1. On the Critical Condition

Soft material wrinkles as a result of mechanical instability. Wrinkles have been well characterized in the study of nonlinear mechanics [28] and are then harnessed as routes in manufacturing tunable surface adhesion [29], photonics [30], bio-mimetic camouflage [31], and acoustic meta-materials [32].

**Figure 9:**
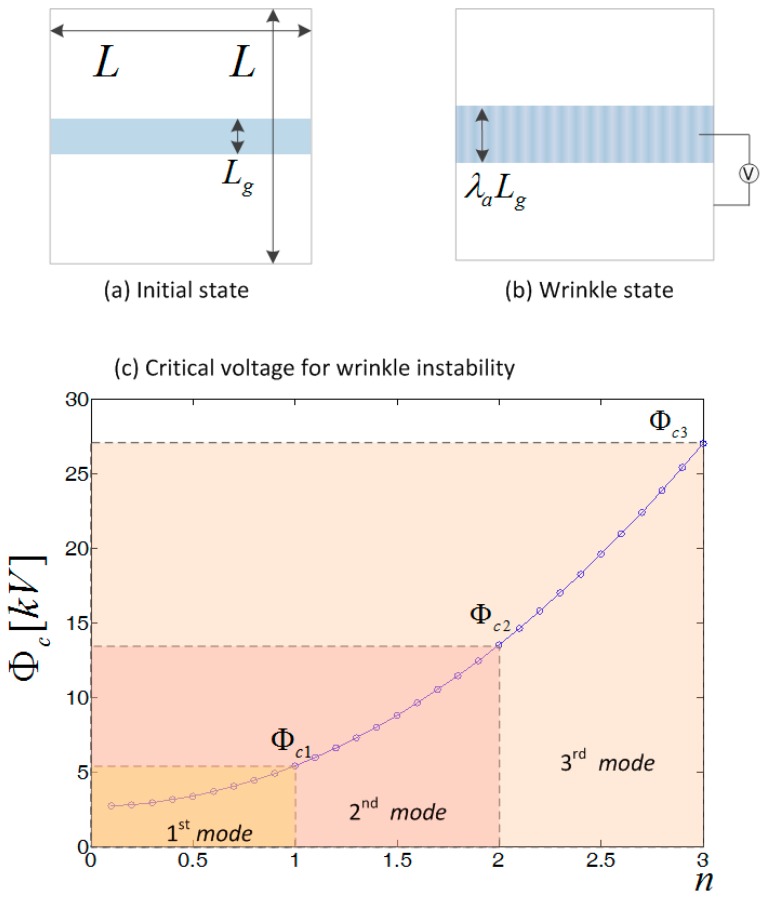
(**a**) The initial state of the hydrogel and dielectric elastomer; (**b**) the wrinkled state in actuation; (**c**) the critical voltage at different order mode of wrinkling instability, where *n* = 1, 2, 3.

Wrinkle instability in hydrogel has been documented and analyzed when the hydrogel swells in solution [33]. So, we used a well-established model with a combination of voltage-triggered instability in dielectric elastomer [34,35]. Figure 9a,b sketches the wrinkled state. Initially, without the voltage, the hydrogel is of the dimension L × $L_{g}$, and at the wrinkled state the dimension changes to L × $\lambda_{a}L_{g}$ where $\lambda_{a}$ represents the actuation stretch. The critical voltage $\Phi_{c}$ on the wrinkle initiation is essential. Herein, due to the negligible inter-facial polarization, the critical voltage in the wrinkle instability is expressed as [34,36]

(2) $$\varepsilon \frac{\Phi_{c}{}^{2}}{H^{2}}∼\frac{\pi E}{12\left(1-E^{2}\right)}{\left(\frac{H}{L\lambda_{a}}\right)}^{2}{\left(1+n^{2}\right)}^{2}$$

where ε is the permittivity, H is the original total thickness of the two hydrogels and the dielectric elastomer before actuation, E is the elastic modulus, ν is the Poisson ratio, $\lambda_{a}$ is the actuation stretch before wrinkling, and n=1,2,3… is the constant.

The value of the parameters is as follows: $\varepsilon =4\times 10^{-11}\;F/m$, $H=0.26\times 10^{-3}\;m$, E=150 kPa, ν=0.49, L=0.06 m, $\lambda_{a}=1.5$ (see the experiments in Figures S3 and S4 of the SM for the material mechanical property). Figure 9c shows the results. Nonlinear mechanical material wrinkles in different instability modes, displaying different patterns on its surface [28]. Hydrogel is able to wrinkle to diverse patterns, including ridge, crease, double period, and crater. Here, in our observation, only the first mode (*n* = 1) sinusoidal pattern was identified with critical voltage (5100 V), which is in agreement with the experiment shown in Figure 5. To trigger the higher order mode of wrinkle, higher voltage is required, which may exceed the electrical breakdown limit in dielectric elastomer, so it is hardly attainable.

### 4.2. Optical Properties

**Figure 10:**
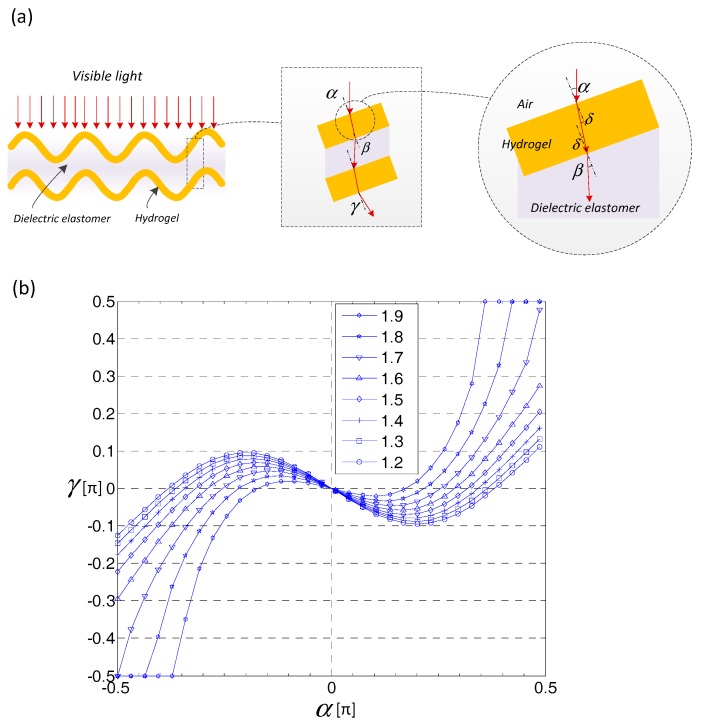
Optical transmission analysis. (**a**) The refraction optical path through the wrinkled hydrogel. (**b**) Calculation of optical excite incident angle.

Figure 10a shows the sketch of the optical transmission through the wrinkled hydrogel and dielectric elastomer. With reference to the experiment, the measurement is illustrated in Figure S5 in SM. Assume that the incident light is at the hydrogel surface position x, with an incident angle α, so the refracted angle γ is expressed by [37]:

(3) $$\gamma =\sin^{-1}\left(\sin\alpha \sqrt{f^{2}-\sin^{2}\alpha }-\frac{1}{2}\sin2\alpha \right)$$

where *f* is the reflected index of the overall hydrogel-elastomer laminate and is taken as 1.2–1.9 [38]. The calculated result is plotted in Figure 10b by varying reflected index, the refracted angle changes, within the range from −π/10 to π/10. Thus, the deeply wrinkled surface is able to absorb more light. This result coincides with a similar blurring actuation in liquid crystal-elastomer hybrid, where the orientation of the liquid crystal is controlled either by the voltage-induced orientation or by the stretching force [39].

## 5. Discussion

In this study, the hydrogel was actuated and wrinkled electrically via a dielectric elastomer. In the study of solid mechanics, when hydrogel has been attached on a thin substrate and wrinkled by compression, this phenomenon is considered as a global instability. The thickness of the two layers is also known to affect the wrinkle profile [40]. In a recent study, thicker hydrogel deforms into double period patterns, which is a surface instability located on the half-infinite substrate [41].

**Figure 11:**
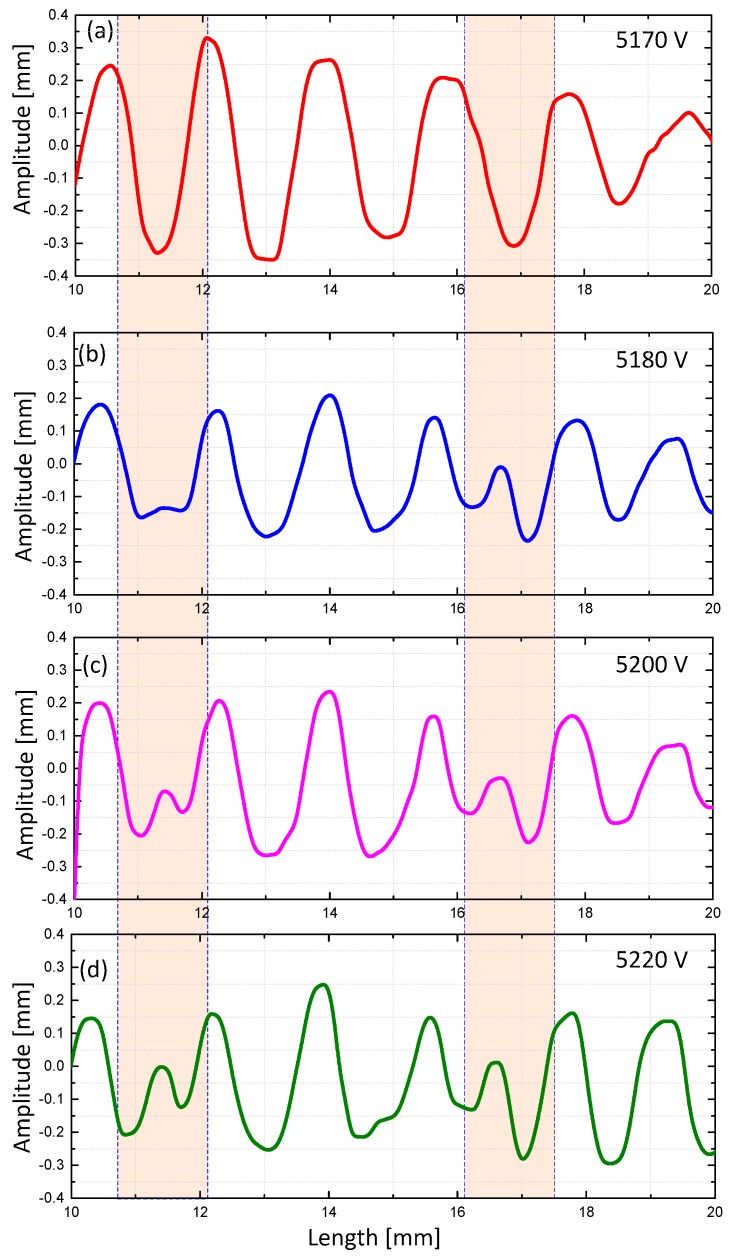
Local growth of a small wrinkle with the voltage at (**a**) 5170 V (**b**) 5180 V (**c**) 5200 V (**d**) 5220 V.

In our experiment, when examining the center part of the hydrogel, we found that with the increment of voltage, one wrinkle period was separated into two small wrinkles, as highlighted in Figure 11. This indicates a similarity in the bifurcation instability [42]. An advanced theoretical study is expected to extend the research of hydrogel actuation and its wrinkle instability.

Reducing the dielectric elastomer actuation voltage is a challenge in application. Current methods include prestretching the membrane, or the use of a thinner film. It is reported that by prestretcing a dielectric elastomer into a thickness level of 14 um, one can lower the actuation voltage into 100 V [43]. This may be employed in the future development of dielectric elastomer-based devices with low energy consumption.

## 6. Conclusions

In summary, a hydrogel was synthesized and actuated electrically using a dielectric elastomer substrate. The hydrogel wrinkles together with the dielectric elastomer, showing an abrupt change in transparency. The electro-wrinkled surface was characterized experimentally. The wavelength and amplitude of the wrinkles were identified at different voltage levels, showing the nonlinear relationship between mechanical deformation and voltage actuation. The change in transparency was measured in the visible light spectrum, illustrating a potential application as a wrinkle-based soft cloaking by a voltage actuation.

## Supplementary Materials

The following are available online at http://www.mdpi.com/2073-4360/10/7/697/s1. Figure S1: The experimental setup for the measurement of the wrinkle. (A) The overall system. (B) The system on the platform. The sample was settled on a manual translation stage. Figure S2: The propagation of the wrinkle through the surface of the hydrogel. Figure S3: Stress-strain curve of the hydrogel electrode. Figure S4: Stress–strain curve of the dielectric elastomer VHB 4910. Figure S5: A sketch of measurement by the spectrophotometer. Figure S6: Blurring effect test (a) a~f were the wrinkled state under the voltage of 4900 V, 5000 V, 5240 V, 5400 V, 5450 V and 5500 V (b) normalized image definition. Video S1: The response of the wrinkling in hydrogel, with respect to a dynamic voltage, of sinusoidal wave of 5200 V amplitude and 1 Hz frequency.
