# Supplementary material for: Voltage-Induced Wrinkle Performance in a Hydrogel by Dielectric Elastomer Actuation
Source: Polymers (Basel). 2018 Jun 22;10(7):697. doi: 10.3390/polym10070697 (PMC6404066; doi:10.3390/polym10070697)
Supplement: Supplementary file 1 [file polymers-10-00697-s001.zip › supplementary materials.pdf]

# SUPPLEMENTARY MATERIALS

## Voltage-induced wrinkle performance in a hydrogel by dielectric elastomer actuation

**Chao Tang[1], Bo Li [1,3]*, Chenbang Zou[1], Lei Liu[1,2], Hualing Chen[1,3,4]**

[1] School of Mechanical Engineering, Xi'an Jiaotong University, Xi'an, Shaanxi 710049, China
[2] State Key Laboratory of Fluid Power and Mechanic Systems, Zhejiang University, Hangzhou, Zhejiang 310027, China China
[3] Shaanxi Province Key Laboratory for Intelligent Robot, Xi'an Jiaotong University, Xi'an, Shaanxi 710049,
[4] State Key Laboratory for Strength and Vibration of Mechanical Structures, Xi'an Jiaotong University, Xi'an, Shaanxi 710049, China

(*) Corresponding Author. Email: liboxjtu@mail.xjtu.edu.cn

## 1. Experimental setup

We measured the out-of-plane displacement of the wrinkle surface as a morphological characterization. The experimental setup is described in Figure S1. A signal generator (No. DG4062, RIGOLTM) and a voltage amplifier (No. 10/10B-HS, Trek AmplifierTM) generate an incremental voltage with a step of 20 V to the dielectric elastomer until the electrical breakdown occurs. The morphology of the wrinkles is characterized by a line laser displacement sensor (LK-G200-1, KeyenceTM) by measuring the out-of-plane deflection of the membrane. The sample is settled at a manual translation stage (TAR-34801, SIGMAKOKI) to measure the displacement in different gel location. The whole wrinkled surface was recorded in the computer.

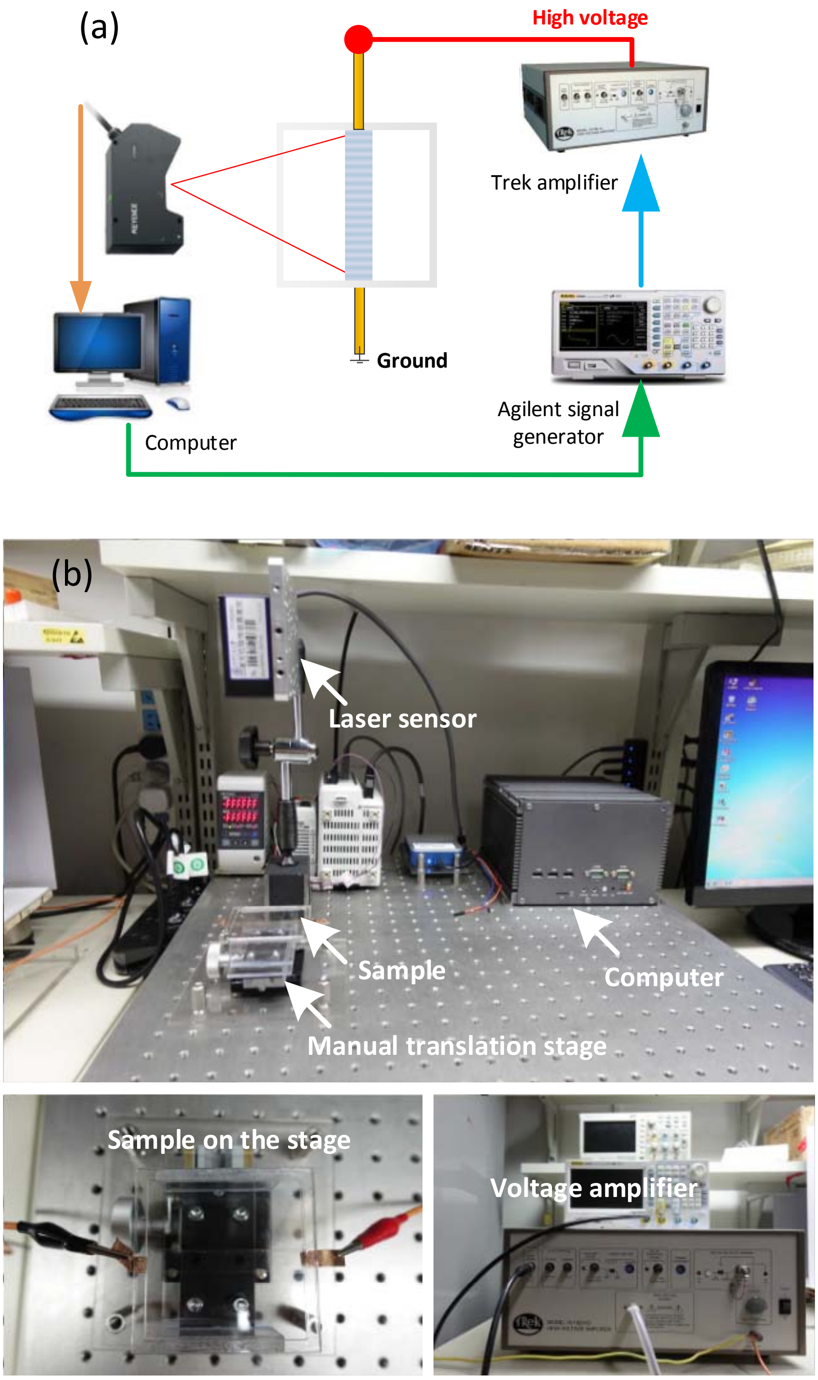


Figure S1. The experimental setup for the measurement of the wrinkle. (a) The overall system. (b) The system on the platform. The sample was settled on a manual translation stage.

## 2. Wrinkle propagation

In the experiments, the wrinkle appeared from the right to the center of the hydrogel, then it propagated to the left by remaining its overall length.

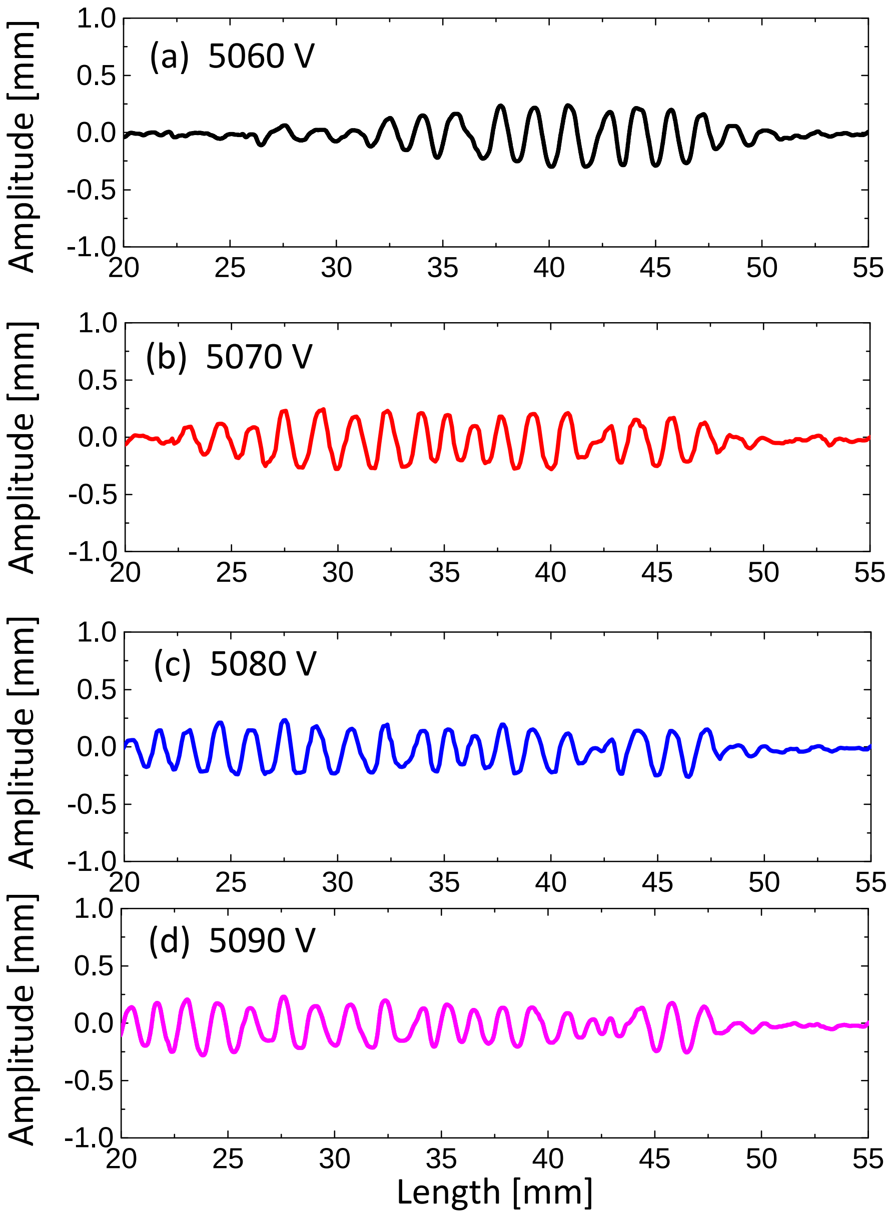


Figure S2. The propagation of the wrinkle through the surface of the hydrogel

## 3. Material parameters

The materials parameters were obtained as follows: the dielectric constant of VHB films is 4.5 [1] and the hydrogel is conductive, so the overall permittivity of the hydrogel/elastomer/hydrogel tri-layer is $4\times10^{-11}$ F/m. The thickness of un-stretch VHB film is 1 mm, and after a 4 × 4 pre-stretch, it is reduced to 0.0625 mm. Thickness of the hydrogel is 0.1mm, so the overall thickness is 0.26 mm.

The elastic moduli of the hydrogel and elastomer were measured by a tensile machine (No. AGS-X, SHIMADZU). The stress-strain curves were plotted in Figure S3 and S4.

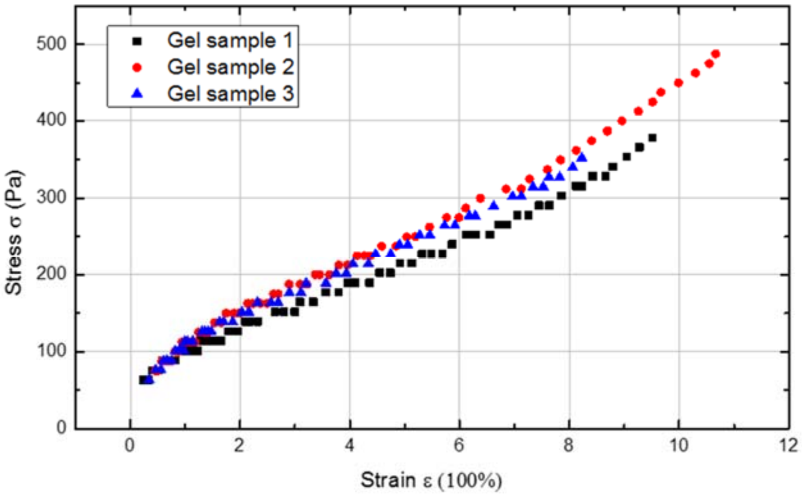


Figure S3. Stress-strain curve of the hydrogel electrode

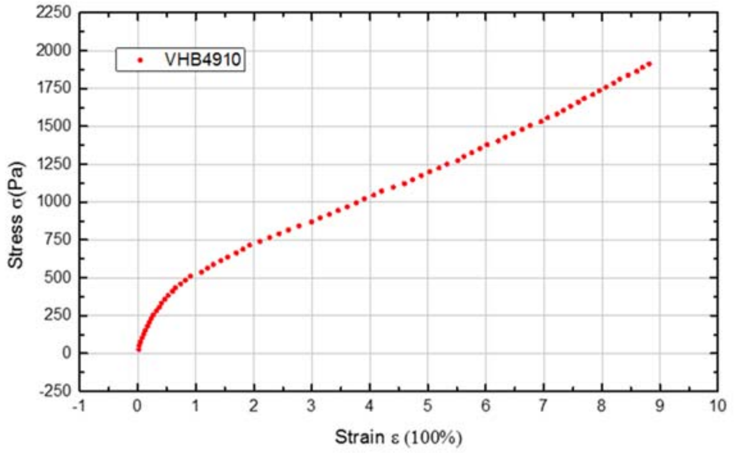


Figure S4. Stress-strain curve of the dielectric elastomer VHB 4910

In our experiments, the deformations of the dielectric elastomer and the hydrogel did not approach the extension limit, thus the neo-Hookean model was adapted in the modeling, and the overall elastic modulus was estimated as 150 kPa.

The Poisson ratio was taken as 0.49 [2]. The actuation stretch was determined by examined the dimension of the hydrogel upon the wrinkling with respect the original dimension through the recorded video, which is $\lambda_a = 1.5$.

4. Optical properties

The transmission spectra was recorded by a spectrophotometer (L2S, Shanghai, INESA). The width of the light spot was able to cover at least one wrinkle wave period and could fully reveal the optical properties, see the illustration in Figure S5.

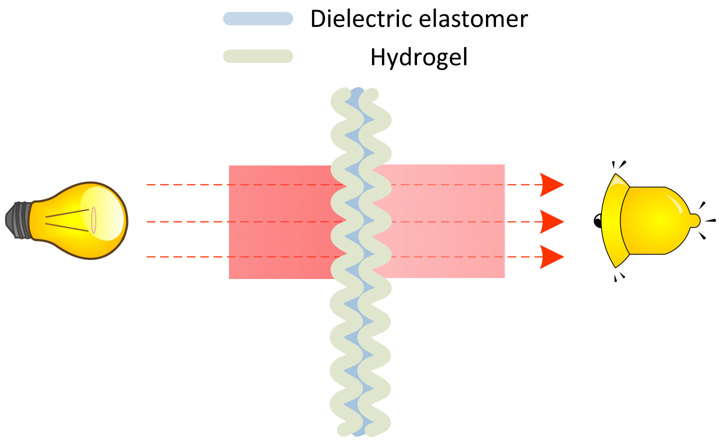


Figure S5. A sketch of measurement in the spectrophotometer

5. Image definition calculation

We test the blurring effect of a bar code when the wrinkle generate. A series of photos were taken by a camera (Sony RX100V) at different voltage as shown in Figure S6 (a).The bar code becomes almost indistinguishable at 5500 V. The image definition was calculated by Matlab R2014a based on the structure similarity (SSIM) [3] algorithm. The effective information of the bar code can be hidden because of the wrinkle, as shown in Figure S6 (b).

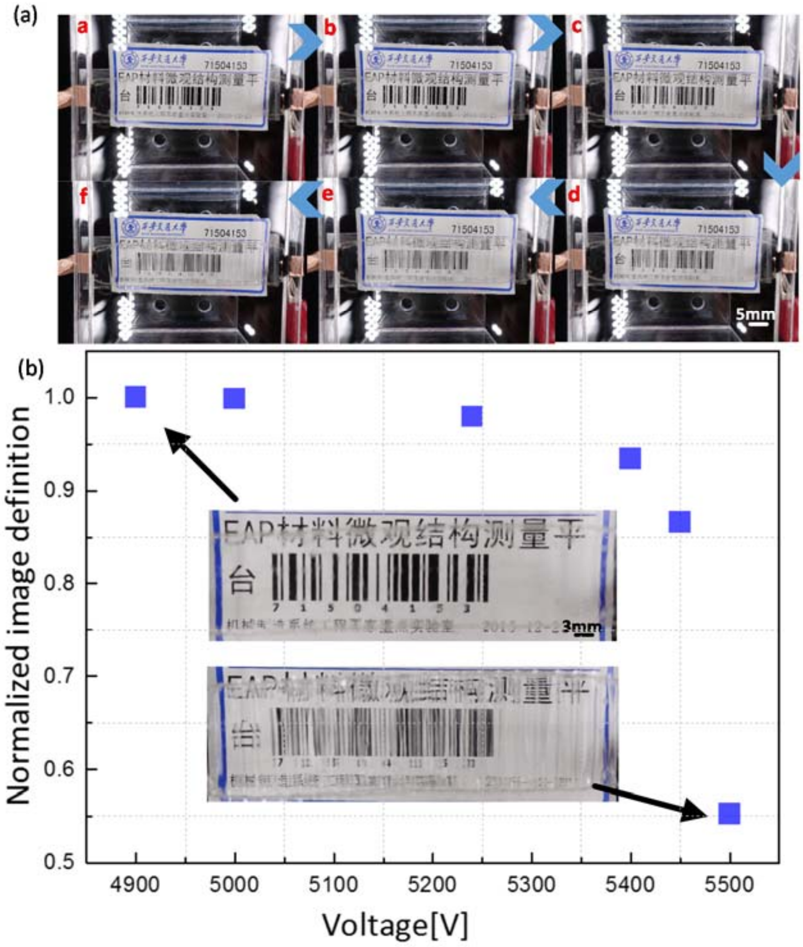


Figure S6. Blurring effect test (a) a~f were the wrinkled state under the voltage of 4900 V, 5000 V, 5240 V, 5400 V, 5450 V and 5500 V (b) normalized image definition

6. Caption of video

Video S1: The response of the wrinkling in hydrogel, with respect to a dynamic voltage, of sinusoidal wave of 5200 V amplitude and 1 Hz frequency.

References

[1] J Qiang,, H. Chen, and B. Li. Experimental study on the dielectric properties of polyacrylate dielectric elastomer [J]. Smart Materials & Structures 21.21(2012):025006.

[2] M. Silvain, et al. A comparison between silicone and acrylic elastomers as dielectric materials in electroactive polymer actuators [J]. Polymer International 59.3(2010):391–399.

[3] Zhou Wang, Bovik, A.C, Sheikh, H.R, et al. Image quality assessment: from error visibility to structural similarity [J]. IEEE Trans Image Process, 2004, 13(4):600-612.
